# Spatiotemporal cluster patterns of hand, foot, and mouth disease at the province level in mainland China, 2011–2018

**DOI:** 10.1371/journal.pone.0270061

**Published:** 2022-08-22

**Authors:** Yuanzhe Wu, Tingwei Wang, Mingyi Zhao, Shumin Dong, Shiwen Wang, Jingcheng Shi

**Affiliations:** 1 School of Civil and Environmental Engineering, University of New South Wales, Sydney, New South Wales, Australia; 2 Xiangya School of Public Health, Central South University, Changsha, Hunan, China; 3 Department of Pediatrics, Central South University Third Xiangya Hospital, Changsha, Hunan, China; 4 School of Technology and Engineering, Fujian Medical University, Fuzhou, Fujian, China; Bangladesh Agricultural University, BANGLADESH

## Abstract

Although three monovalent EV-A71 vaccines have been launched in mainland China since 2016, hand, foot, and mouth disease (HFMD) still causes a considerable disease burden in China. Vaccines’ use may change the epidemiological characters of HFMD. Spatial autocorrelation analysis and space-time scan statistics analysis were used to explore the spatiotemporal distribution pattern of this disease at the provincial level in mainland China. The effects of meteorological factors, socio-economic factors, and health resources on HFMD incidence were analyzed using Geodetector. Interrupted time series (ITS) was used to analyze the impact of the EV-A71 vaccine on the incidence of HFMD. This study found that the median annual incidence of HFMD was 153.78 per 100,000 (ranging from 120.79 to 205.06) in mainland China from 2011 to 2018. Two peaks of infections were observed per year. Children 5 years and under were the main morbid population. The spatial distribution of HFMD was presented a significant clustering pattern in each year (P<0.001). The distribution of HFMD cases was clustered in time and space. The range of cluster time was between April and October. The most likely cluster appeared in the southern coastal provinces (Guangxi, Guangdong, Hainan) from 2011 to 2017 and in the eastern coastal provinces (Shanghai, Jiangsu, Zhejiang) in 2018. The spatial heterogeneity of HFMD incidence could be attributed to meteorological factors, socioeconomic factors, and health resource. After introducing the EV-A71 vaccine, the instantaneous level of HFMD incidence decreased at the national level, and HFMD incidence trended downward in the southern coastal provinces and increased in the eastern coastal provinces. The prevention and control policies of HFMD should be adapted to local conditions in different provinces. It is necessary to advance the EV-A71 vaccination plan, expand the vaccine coverage and develop multivalent HFMD vaccines as soon as possible.

## Introduction

Hand, foot, and mouth disease (HFMD) is a common infectious disease in childhood caused by various enteroviruses. The most commonly reported pathogens are Enterovirus A71 (EV-A71) and Coxsackievirus A16 (CV-A16) [1]. HFMD can be transmitted through close contact with virus-contaminated objects or contaminated respiratory droplets, water, and food [2]. Symptoms of HFMD typically include flu-like infections, skin eruptions on hands and feet, and oral herpes [3, 4]. Most patients show mild and self-limiting symptoms, while few patients may occur complications such as myocarditis, pulmonary edema, and aseptic meningitis. Some severe cases will develop rapidly and eventually death [5]. HFMD has a severe impact on children’s health under five years [1, 5].

In the past two decades, some Asian countries frequently reported outbreaks of HFMD, which has become a significant public health problem across the Asia-Pacific region [6–9]. HFMD is prevalent throughout China as a perennial disease [2]. By 2018, the Chinese Center for Disease Control and Prevention (Chinese CDC) had reported 20.5 million cases of HFMD, including 3,667 deaths. HFMD has caused an enormous burden of disease in China [10]. In May 2008, HFMD was classified as a category C notifiable infection in China. Medical institutions have been required to report it through the Notifiable Infectious Disease Reporting System (NIDRIS) within 24 hours [11].

Affected by factors such as average temperature, average relative humidity, monthly precipitation, population density, environmental sanitation, etc., the spatial distribution of the HFMD incidence shows differences [12]. From the perspective of temporal dimension, the peak of HFMD occurs in summer in temperate regions, but in the tropical areas, HFMD can occur at any time of the year [13]. Some researchers have found evidence of spatial distribution patterns and spatiotemporal clusters of HFMD [14, 15]. Analyzing the spatiotemporal cluster patterns of infections can contribute to identifying high-risk areas and populations, exploring disease risk factors, formulating effective measures to prevent and control the spread of diseases, and allocating public health resources reasonably [15, 16]. Because the risk of HFMD varies with space and time, it is imperative to determine the spatiotemporal clusters of HFMD in China [17].

Some studies have shown that latitude will affect the incidence of HFMD to a certain extent, and the incidence of HFMD is higher in low latitudes such as tropical and temperate regions [18]. Mainland China spans multiple temperature zones. The incidence of HFMD in southern China is higher than that in the north, and there are two peaks (summer and autumn) of HFMD each year in southern China, while there is only one peak(summer) each year in northern China [1]. Natural and social factors vary greatly between the south and north, coastal and inland areas, and the prevalence patterns of HFMD are different in different regions [18, 19]. Previous studies have found that precipitation, relative humidity, temperature, gross domestic products (GDP), and the population density are the main factors affecting the incidence of HFMD in China [20–22]. Most of the studies were conducted in a single province or local area, while the influence of meteorological and socioeconomic factors is more complex in the vast area of mainland China. In addition, a study found that the number of hospital beds per capita was also one of the dominant determinants that influence HFMD incidence in Shandong [23]. The number of hospital beds per capita and the number of health technicians per capita are critical indicators to measure the health resources of a region, but most studies ignored the influence of this factor. Quantifying the relationship between driving factors and HFMD incidence in the whole of mainland China can provide a theoretical basis for the macro prevention policy of HFMD.

From 2008 to 2012, China had reported 7.2 million cases of HFMD, of which were 2457 fatal [1]. Several studies had reported that HFMD spread in the complex space-time domain, and the space-time cluster was detected in mainland China from 2008 to 2012 [16, 24]. Since 2016, three inactive monovalent EV-A71 vaccines have been launched in China, which may change the epidemiological characters of HFMD. A study in Guangxi found that the incidence, mortality rate, and case-severity rate of HFMD decreased with years after the introduction of the EV-A71 vaccination. The proportion of HFMD cases aged 0–12 months decreased and there were more HFMD cases occurred in rural areas compared with before the introduction of the EV-A71 vaccination [25]. Since then, researches on the spatial and temporal distribution pattern of HFMD has mainly been concentrated in several provinces and cities, such as Shandong [26], Xinjiang [19], Shaanxi [27]. However, few studies have been conducted in the whole of China or have analyzed the actual impact of the introduction of the HFMD vaccine on HFMD incidence. Determining the spatiotemporal cluster variation of HFMD after the vaccine was launched will help define new high-risk areas and periods. Interrupted time series (ITS) analysis is increasingly being used to assess the effectiveness of population-level health interventions implemented at a clearly defined point in time [28]. It can be used to understand the impact of the EV-A71 vaccine on the incidence of HFMD in mainland China. The combination of space-time scan statistics analysis and interrupted time series can provide a basis for relevant departments to implement disease control and prevention policies in key areas.

Therefore, this study was aimed to 1) analyze the spatiotemporal cluster of HFMD at the province level in mainland China from 2011 to 2018, compare the difference before and after the vaccines were launched, 2) quantify the relationship between meteorological, socio-economic factors, health resources and HFMD incidence, and 3) analyze the actual impact of the introduction of the HFMD vaccine on HFMD incidence.

## Methods

### Data resources and study area

All HFMD cases’ data were obtained from the public health science data center given by the Chinese Center for Diseases Control and Prevention (Chinese CDC) [29]. All HFMD cases were diagnosed according to the unified diagnostic criteria issued by the Ministry of Health of China and reported to the infectious disease surveillance system [2]. This HFMD cases database collected all the data reported since the direct online reporting from 2008. The main content included the number of cases and death, incidence and mortality by region, age group, and gender [29]. The incidence of HFMD was calculated as the ratio of the number of cases to the number of permanent residents during the study period. The population data from 2011 to 2015 were collected from Chinese CDC Infectious Disease Network reports population data, which was based on the population data released by the National Bureau of Statistics and determined by the provinces after checking and adjusting. Due to the lack of data from 2016 to 2018, the remaining data were collected from the National Bureau of Statistics of China [30].

The monthly meteorological data for the same period was obtained from the National Meteorological Information Center [31], including the average temperature, relative humidity, precipitation, and hours of sunlight of each province (or autonomous region, municipalities). The provincial socioeconomic data and health resource data were obtained from the National Bureau of Statistics of China [30], including gross domestic products (GDP) per capita, population density, hospital beds per 10,000 persons, and health technicians per 10,000 persons.

Because this HFMD cases database did not contain data on HFMD cases in Taiwan, Hong Kong, and Macau, the actual study area was mainland China, which includes 22 provinces, 4 municipalities, and 5 autonomous regions, a total of 31 provincial-level administrative regions (excluding Hong Kong, Macau and Taiwan). The national 1:42,000,000 administrative division map(examination number: GS (2019) 1655), which was provided by the Standard Map Service System, was used as the base map to establish a vector layer of administrative boundaries in units of provinces (autonomous regions, municipalities directly under the Central Government) [32].

A total of 17,118,050 cases of HFMD were included in this study, which was reported by 31 provincial administrative regions in Mainland China from January 2011 to December 2018. Microsoft Excel (version 2013, Microsoft Corp, Redmond,WA, USA) was used to build the database, and the incidence of each local administrative region each year was represented in different colors on the map by ArcMap (version 10.2, ESRI Inc., Redlands, CA, USA).

### Spatial autocorrelation analysis

#### Global spatial autocorrelation analysis

The global spatial autocorrelation analysis started from the macro-level of the whole country, compared the mean value of the attribute in the general area and the attribute value on each spatial unit to obtain the average degree of association between the incidence of HFMD in various provinces across the country. That was, to determine whether the incidence of HFMD was clustered nationwide. Global Moran’s *I* is a commonly used global correlation index ranging from -1 to 1 [33]. *I*> 0 indicates a positive spatial correlation at the given significance level. The larger the value, the more pronounced the spatial correlation, high observations accumulate in space with high observations, and low observations accumulate spatially. *I* <0 indicates a negative spatial correlation. The smaller the value, the more significant the spatial difference, the tendency of high observations to cluster with low observations. If *I* = 0, the observations are randomly distributed in space [34].

#### Local spatial autocorrelation analysis

Local indicator of spatial association (LISA) is the analysis index of local spatial autocorrelation analysis [35]. In this study, it was used to measure the degree of spatial dependence between the HFMD incidence of a province and the HFMD incidence of its neighboring provinces and identify its spatial cluster pattern in a local space. There are four spatial correlation patterns: High-High cluster, Low-Low cluster, High-Low cluster, and Low-High cluster. High-High cluster and Low-Low cluster indicate that the observations have a strong positive spatial correlation. The two patterns respectively indicate that the high-incidence areas are adjacent to the high-incidence areas, and the low-incidence areas are adjacent to the low-incidence areas. The High-Low cluster and Low-High cluster signify that the observations have a strong negative spatial correlation. The two patterns mean that high-incidence areas are adjacent to low-incidence areas.

GeoDa (version 1.16.016, GitHub, San Francisco, CA, USA) was used for spatial autocorrelation analysis, and the results of local spatial autocorrelation were displayed on the map with ArcMap (version 10.2, ESRI Inc., Redlands, CA, USA).

#### Space-time scan statistics analysis

The space-time scan statistics analysis was proposed by Kulldorff, which can determine the space-time cluster [36]. The principle is to establish a scanning window that moves in space, the radius of the window is constantly changing according to the population of the study area, and an infinite number of circular windows with different radius are generated to detect possible spatial agglomeration areas. Calculate the expected number of cases based on the total incidence and the population in each scanning window, and then use the ratio of the actual number of cases inside and outside the window to the expected number of cases to calculate the relative risk (RR) and log-likelihood ratio (LLR). The window with the maximum LLR was defined as the most likely cluster, and other windows with smaller LLR, which were statistically significant, were defined as secondary clusters. The Markov Chain Monte Carlo (MCMC) method generates 999 simulation data sets, obtaining the probability P-value. The formula of *LLR* is:

$$LLR=\text{ln}\frac{L\left(z\right)}{L_{0}}=\text{ln}{\left(\frac{c}{n}\right)}^{c}{\left(\frac{C-c}{C-n}\right)}^{C-c}$$

*L*_*0*_ is the likelihood under the null hypothesis, which is always a constant for a certain data set, *L(z)* is the likelihood of a certain area under the alternative hypothesis, c is the actual number of cases in the window, and n is the window under the null hypothesis Expected number of cases, *C* is the total number of cases, *C-c* and *C-n* are the actual number of cases outside the window and the number of expected cases, respectively [36, 37].

The space-time scan statistics analysis results are very sensitive to the parameter settings of the maximum spatial cluster size and the maximum length of the temporal scanning window [38]. Hjalmars suggested that 10% of the population at risk should be selected as the size of the maximum cluster [39]. A study recommended 15% of the number of people at risk as to the appropriate scale for the largest spatial scanning window [40]. In addition, the coverage of the largest cluster should not exceed 15% of the study area [41]. Across the country, HFMD had a peak activity in half a year. Southern China had two peak outbreaks of HFMD each year, and northern China had one peak outbreak each year [1]. Therefore, this study set 15% of the total population as the maximum cluster size, and the time frame of the scan analysis was set to one year to observe the cluster changes during the study.

SaTScan (version 10.0, Martin Kulldorff, Harvard Medical School, Boston, MA, USA and Information Management Services Inc, Calverton, MD, USA) was used to perform space-time scan statistics analysis of HFMD cases in 31 provinces of mainland China from 2011 to 2018. The analysis results were visualized on the map with ArcMap (version 10.2, ESRI Inc., Redlands, CA, USA).

### Geographical Detector

Geographical Detector (Geodetector) is a set of statistical methods for detecting Spatial Stratified Heterogeneity (SSH) and uncovering the driving forces behind it [42]. Its core idea is based on the assumption that if an independent variable has an important influence on a dependent variable, the spatial distribution of the independent variable and the dependent variable should be similar [43]. The Geodetector requires the independent variable to be categorical, and if the independent variable is a continuous numerical variable, it should be stratified. The natural breakpoint method minimizes the data difference in the same category and maximizes the difference between categories. This study adopted the natural breakpoint method to stratify the data, using the 8 layers with the highest explanatory power.

The Geodetector consists of risk detector, factor detector, ecological detector, and interaction detector. In this study, we mainly applied the factor detector and the interaction detector. The Factor detector was used to calculate the explanatory power of each influencing factor on the spatial differentiation of HFMD. The explanatory power for the incidence of HFMD was calculated by using the sum of variances within the layer and the total variance of the whole region to determine the main factors affecting HFMD. The explanatory power is measured by *q*, and the formula for calculating *q* is

$$q=1-\frac{\sum_{h=1}^{L}N_{h}\sigma_{h}^{2}}{N\sigma^{2}}=1-\frac{SSW}{SST}$$

$$SSW=\overset{L}{\underset{h=1}{\sum }}N_{h}\sigma_{h}^{2},\;SST=N\sigma^{2}$$

Where q represents the explanatory power of a driving factor on HFMD spatial differentiation. The value of *q* ∈ [0,1]. The larger the value of *q*, the stronger the explanatory power of this factor for HFMD differentiation will be. Where *h* is the number of layers of the dependent variable *Y* or independent variable *X*. In this paper, the number of layers is 8. *N*_*h*_ and *N* are the number of units in layer *h* and in the whole study region, respectively. *σ*^2^ is the variance of *Y* in the whole study region, and $\sigma_{h}^{2}$ is the variance of *Y* in layer *h*. SSW and SST are the sum of variance in all layers and total variance of the whole study region, respectively.

The interaction detector was used to identify the interactions between different influencing factors *X*_1_ and *X*_2_. It shows whether the interaction will increase or decrease the explanatory power of the dependent variable *Y*. The *q*-value of factors *X*_1_ and *X*_2_ were recorded as *q* (*X*_1_) and *q* (*X*_2_) in the factor detector. The two factors were superimposed and computed a new *q*-value as *q*(*X*_1_ ∩ *X*_2_) in the interaction detector. By comparing the value of *q* (*X*_1_), *q* (*X*_2_) and *q*(*X*_1_ ∩ *X*_2_), we can determine the influence of the interaction. The interaction relationships are cataloged as follows:

Nonlinear weakening: if *q*(*X*_1_ ∩ *X*_2_) < *Min*(*q*(*X*_1_), *q*(*X*_2_))
Single-factor nonlinear weakening: if *Min*(*q*(*X*_1_), *q*(*X*_2_)) < *q*(*X*_1_ ∩ *X*_2_) *Max*(*q*(*X*_1_), *q*(*X*_2_))
Double-factor enhancement: if *q*(*X*_1_ ∩ *X*_2_) > *Max*(*q*(*X*_1_), *q*(*X*_2_))
Independent: if *q*(*X*_1_ ∩ *X*_2_) = *q*(*X*_1_) + *q*(*X*_2_)
Nonlinear enhancement: if *q*(*X*_1_ ∩ *X*_2_) > *q*(*X*_1_) + *q*(*X*_2_)
Geodetector can be implemented through Geodetector software, it is freely available from http://www.geodetector.cn/ [44].

### Interrupted time series analysis

In this study, the interruption time series analysis took the time when the EV-A71 vaccine was officially introduced for vaccination as the intervention point, divided into a pre-intervention phase and a post-intervention phase. The introduction time of vaccines in each province was determined according to the documents or news on the official websites of each province’s CDC. The basic model was set as:

$$Y=\;\beta_{0}+\beta_{1}\times time+\beta_{2}\times intervention+\beta_{3}\times posttime+\beta_{4}\times C+\varepsilon$$

*Time* is a time count variable, the number of months from the first observation point to a follow-up, *time* = 1, 2, 3,……, n, n is the number of observations points; *intervention* is an intervention indicator variable, the value of the observation point before the intervention is 0, and the value of the observation point after the intervention is 1; *posttime* is the time counting variable after the intervention, the *posttime* of the observation point before the intervention is 0, the *posttime* of the first month after the intervention is 1, and so on; *C* is other control variables, such as seasonal trends; *ε* represents the variation not explained by the model. The coefficient *β*_0_ represents the baseline level at *time* = 0, *β*_1_ indicates the change in outcome related to the increase in time unit (representing the change trend of the outcome variable before the intervention), *β*_2_ represents the instantaneous change of the outcome variable caused by the intervention (the amount of immediate level change), *β*_3_ indicates the amount of change in the trend of the outcome variable after the intervention. In this study, the outcome variable *Y* is the number of hand, foot, and mouth disease cases (count data). We used the Poisson regression model and population (log-transformed) as an offset variable in order to transform back to rates. We used the Fourier term to adjust for seasonality and Prais-Winsten to adjust for autocorrelation.

R Studio open source (version 2021.09.1+372, rstudio, Boston, MA, USA) was used to build the model and visualize the results.

## Result

### Epidemiology characteristics of HFMD cases

17,118,050 HFMD cases were reported from 2011 to 2018 in mainland China, with a median annual incidence of 153.78 per 100,000 (ranging from 120.79 to 205.06). A total of 2283 cases died with a case fatality rate of 0.13‰. Children 5 years and under were the main morbid population, accounting for 94.26% of all reported cases. The results of the reported cases grouped by age are shown in Table 1. In 2018, the incidence of children who are 5 years and under was 92.76%, the lowest level in 8 years.

**Table 1:** Distribution characteristics of HFMD cases by age group in Mainland China, 2011–2018 [n (%)].

Age (year)	Total	2011	2012	2013	2014	2015	2016	2017	2018
**<1**	1690132(9.87)	143335(8.85)	203518(9.38)	238467(13.04)	215022(7.74)	225568(11.29)	211015(8.64)	232110(12.03)	221097(9.40)
**1~**	5424410(31.69)	473667(29.24)	634419(29.25)	647868(35.43)	849911(30.58)	685132(34.30)	745728(30.54)	615872(31.92)	771813(32.80)
**2~**	3740821(21.85)	397579(24.55)	514655(23.73)	392159(21.45)	660375(23.76)	432349(21.65)	520068(21.30)	379502(19.67)	444144(18.87)
**3~**	2913215(17.02)	296888(18.33)	388539(17.92)	268944(14.71)	482998(17.38)	325139(16.28)	445141(18.23)	317762(16.47)	387804(16.48)
**4~**	1591716(9.30)	152038(9.39)	207850(9.58)	132113(7.23)	271527(9.77)	154119(7.72)	258059(10.57)	180082(9.33)	235928(10.03)
**5~**	775163 (4.53)	71436 (4.41)	98047 (4.52)	63279 (3.46)	135197(4.87)	75674(3.79)	117945(4.83)	91490(4.74)	122095(5.19)
**>5**	982593 (5.74)	84763 (5.23)	121709(5.61)	85547 (4.68)	163831(5.90)	99390 (4.98)	144192(5.90)	112732(5.84)	170429(7.24)

**Fig 1:**
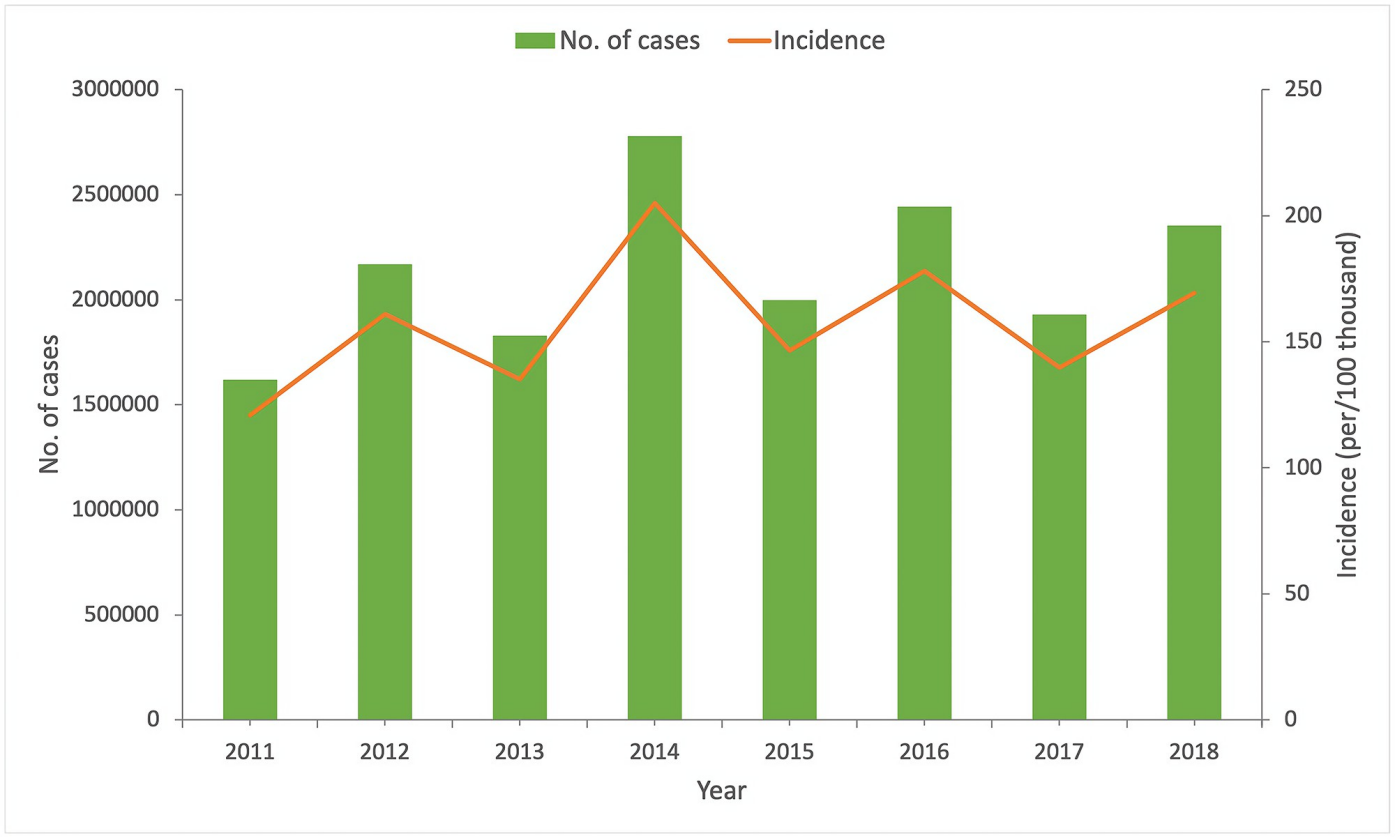
Annual incidence and number of cases of HFMD in mainland China,2011–2018.

**Fig 2:**
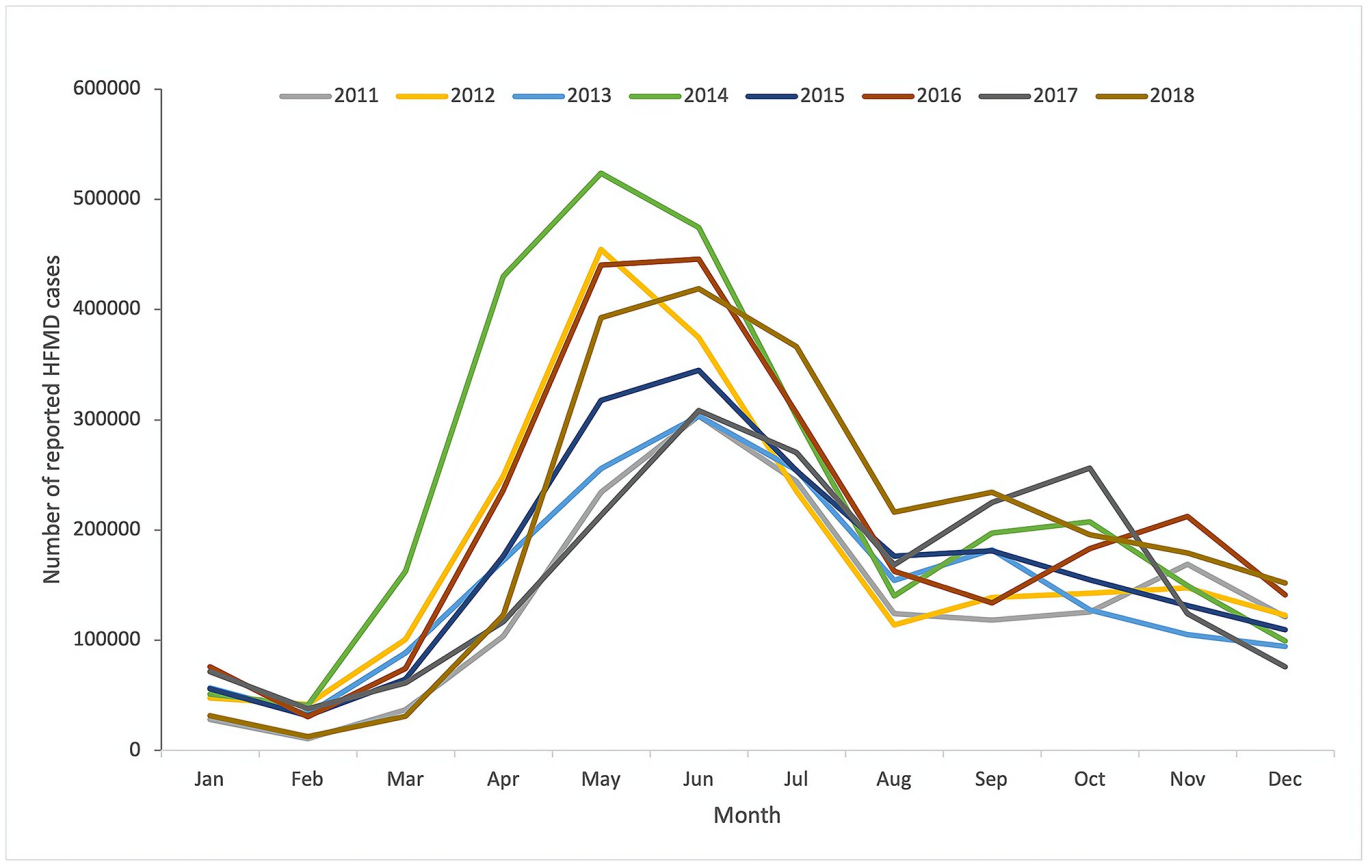
Monthly distribution of HFMD cases in mainland China, 2011–2018.

Fig 1 shows the epidemic trend of the number of reported cases and annual incidence of HFMD in mainland China from 2011 to 2018. The number of reported cases and annual incidence of HFMD increased in even-numbered years (i.e.2012, 2014, 2016, 2018) and declined in odd-numbered years (i.e.2011, 2013, 2015, 2017) in mainland China, which demonstrated a phenomenon of increasing incidence in a two-year cycle from 2011 to 2014 and slightly decreasing incidence in a two-year cycle from 2015 to 2018. The incidence of HFMD peaked in 2014. Fig 2 shows the monthly distribution of the reported cases of HFMD in Mainland China from 2011 to 2018. Two peaks of infections were observed per year. The first peaks occurred from April to July, and the second occurred from September to November. The first peaks were higher than the second over the entire investigated period. In general, the result indicated that the incidence of HFMD was potentially seasonal and cyclical.

**Fig 3:**
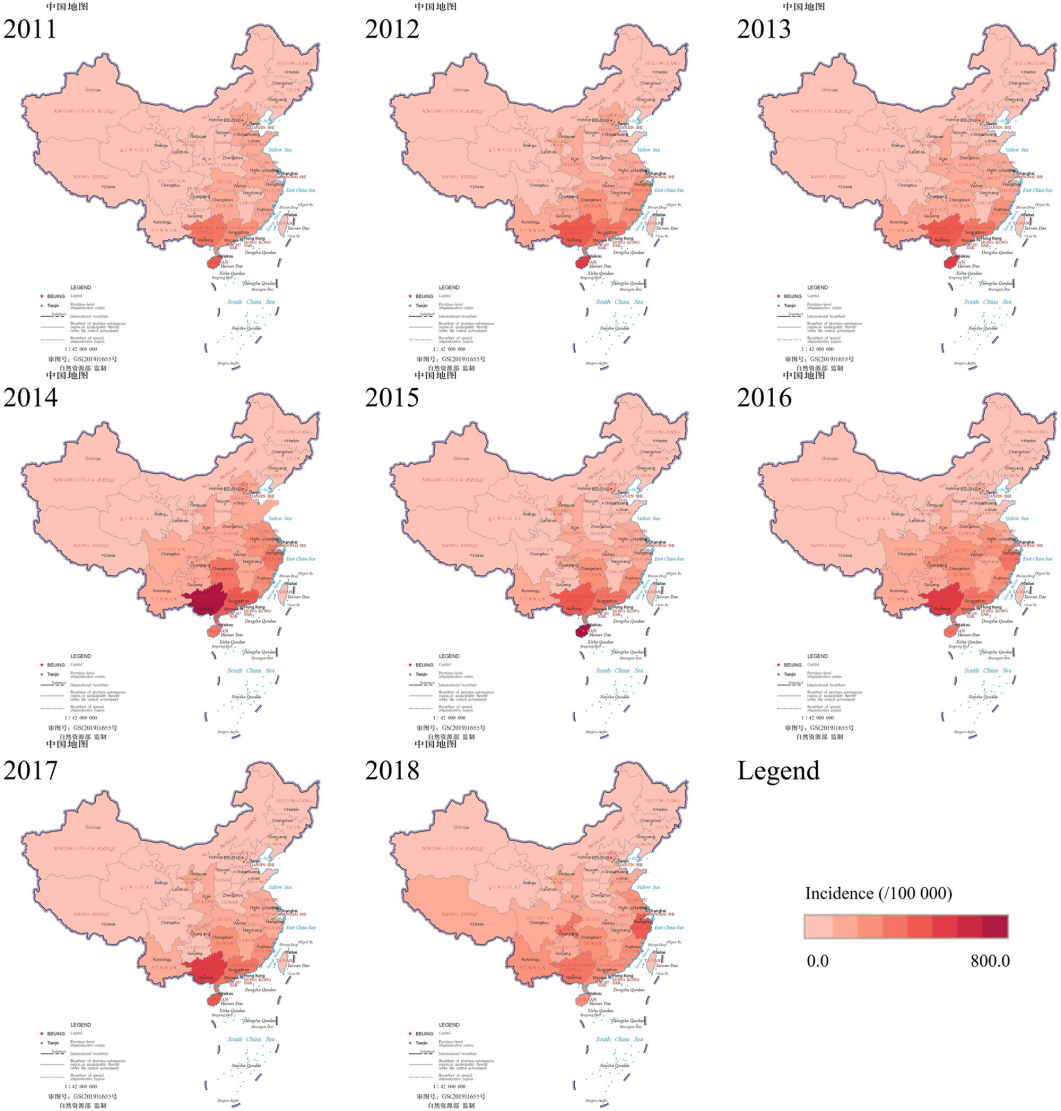
Spatial distribution of HFMD incidence in mainland China, 2011–2018.

The spatial distribution of the incidence of HFMD in mainland China is shown in Fig 3, and dark color indicates high incidence. The incidence of HFMD varied greatly among provinces. The incidence was higher in eastern and southern mainland China than in western and northeastern regions. The incidence of HFMD in the southern coastal provinces (Hainan, Guangxi, Guangdong) had been at the forefront of mainland China for many years. The inland province of Hunan had also been at a high incidence level for many years. Zhejiang had the highest incidence of HFMD in 2018. Adjacent to Zhejiang, Jiangsu, Shanghai, and Fujian also had high incidences. The western province of Chongqing showed the second-highest incidence this year. This result indicates the expansion of high-incidence areas.

### Spatial autocorrelation analysis

The results of global spatial autocorrelation are shown in Table 2. The province-level of mainland China had a high global spatial autocorrelation from 2011 to 2018(global Moran’s I > 0.419, *P* < 0.001). This result indicated that the spatial distribution of HFMD cases was non-random in mainland China, and there was a statistically significant positive correlation.

**Table 2:** Results of the global spatial autocorrelation analysis on HFMD incidence in Mainland China, 2009–2018.

Year	Moran’s I	Z-Value	*P*-Value
2011	0.419	4.778	0.001
2012	0.500	4.868	0.001
2013	0.485	4.857	0.001
2014	0.476	4.581	0.001
2015	0.432	4.899	0.001
2016	0.503	4.694	0.001
2017	0.547	5.386	0.001
2018	0.534	4.664	0.001

**Fig 4:**
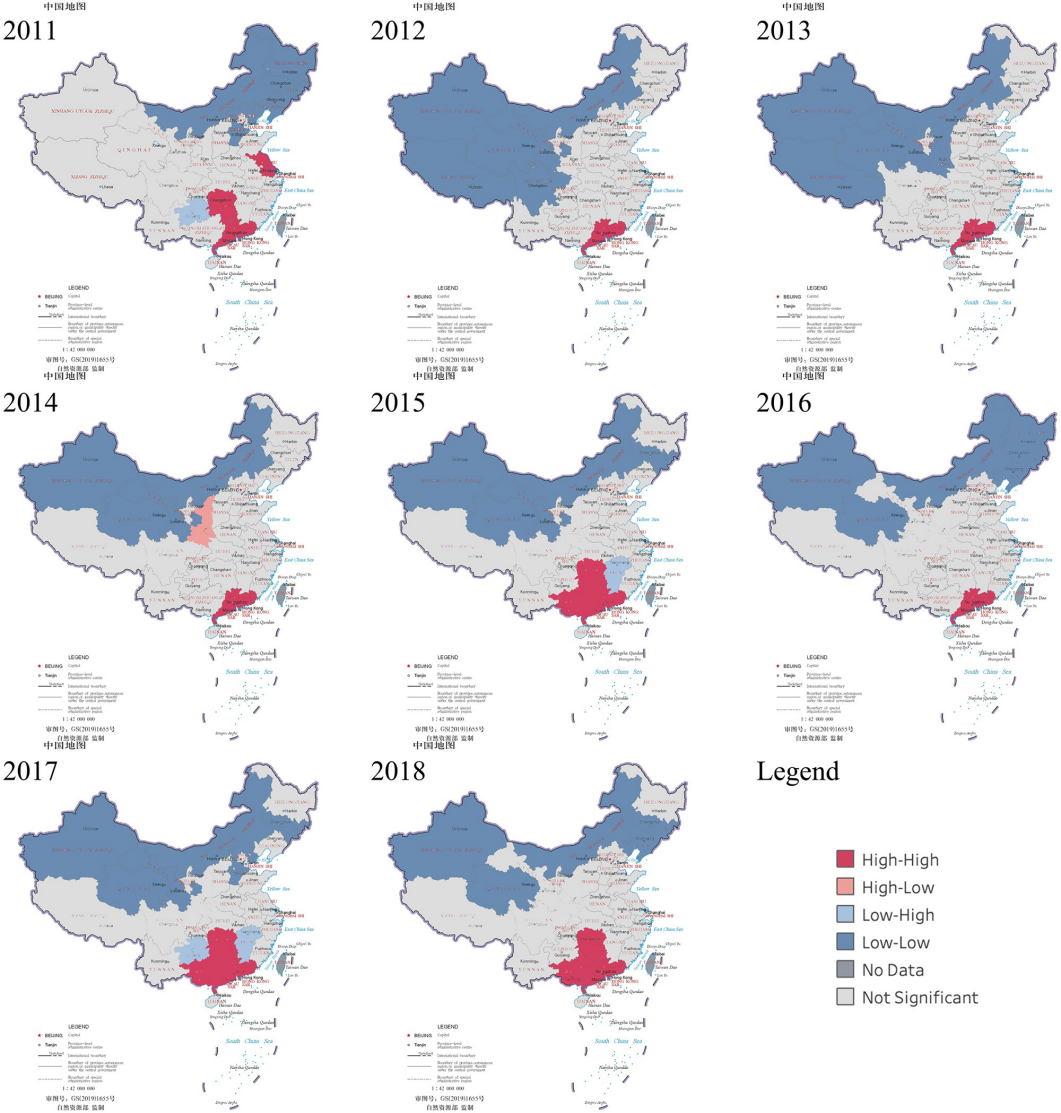
Spatial correlation cluster maps of HFMD incidence in mainland China, 2011–2018.

According to the result of local spatial autocorrelation analysis combined with the LISA value, there were spatial correlation cluster maps for HFMD incidence in mainland China from 2011 to 2018 (Fig 4). In this study, positive spatial association (High-High cluster and Low-Low cluster) was found in mainland China every year from 2011 to 2018. High-High clusters mainly appeared in a few provinces in southern China, including Guangdong, Guangxi, and Hunan. The results indicated that these provinces and their neighboring provinces have a high incidence of HFMD. The High-High cluster was found in new regions (Zhejiang and Fujian) in 2018, which meant that areas with a high incidence of HFMD have expanded. Low-Low cluster was only found in western and northern China, mainly Xinjiang, Xizang, Qinghai, and NeiMongol, which indicated that the incidence of HFMD is relatively low in western and northern China. Negative spatial associations (High-Low cluster and Low-High cluster) were scattered in mainland China and only found in a few inland provinces in a few years.

### Spatiotemporal clusters analysis

**Fig 5:**
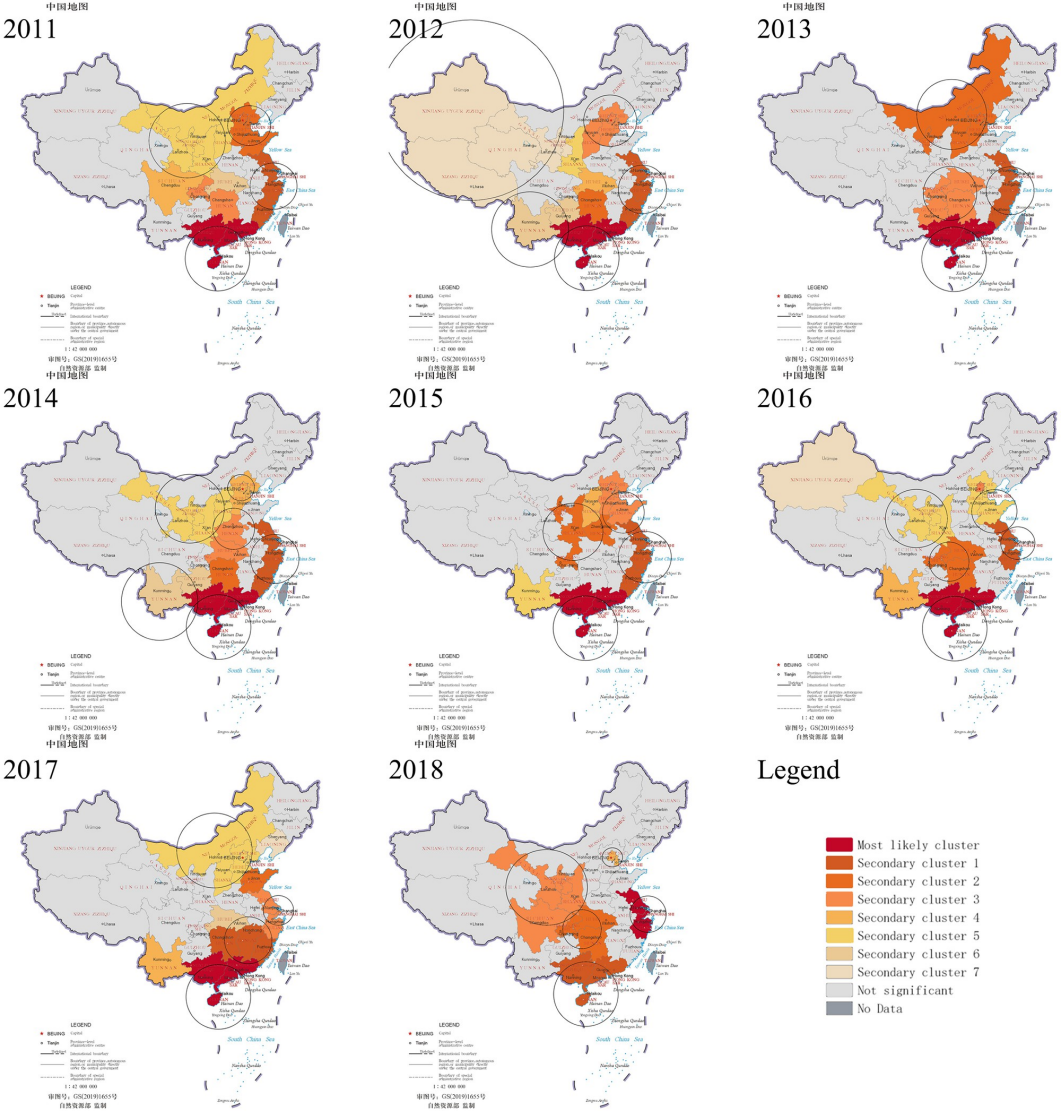
Spatiotemporal clusters of HFMD in mainland China, 2011–2018.

Space-time scan analysis of HFMD data was conducted year by year in mainland China from 2011 to 2018. The results showed that the cases of HFMD were not randomly distributed in mainland China during the study period. Each year at least five significant spatiotemporal clusters were found, including the most likely cluster and secondary clusters. The red part on the map is the area of the most likely cluster (Fig 5).

Table 3 shows the cluster time, cluster province, relative risk, and other information of the most likely cluster from 2011 to 2018. In most years (2011–2017), the most likely cluster was in the southern coastal provinces, including Hainan, Guangxi, and Guangdong. The range of cluster time for these years was between April and October. The relative risk ranged from 3.75 to 6.53, indicating that the incidence risk in the clusters was 3.75 to 6.53 times that of outside the gathering area in these years. The most likely cluster was different from the past in 2018, which appeared in the eastern coastal areas, including Shanghai, Jiangsu, and Zhejiang. The cluster time was from May to September, and the relative risk was 3.45. Summarizing the results of space-time scan analysis from 2011 to 2018, we observed that the risk areas for HFMD were mainly southern coastal provinces (Hainan, Guangxi, Guangdong), followed by eastern coastal provinces (Zhejiang, Shanghai, Jiangsu, Fujian), which with a developed economy, high population density and strong population mobility. The high-risk time frame was at the end of spring, throughout the summer, and early autumn, when the climate is warm and humid. Although the central and southwest regions were not included in the most likely cluster, their relative risk was at the forefront for many years, and these provinces need to be paid attention to.

**Table 3:** The most likely spatiotemporal clusters of HFMD in mainland China, 2011–2018.

Scan Timeframe	Cluster Time	Cluster Province	Observed Cases	Expected Cases	Relative Risk	*P*-value
2011	May.1 ~ Oct.31	Hainan, Guangxi, Guangdong	368381	114465.30	3.75	<0.01
2012	Apr.1 ~ Sept.30	Hainan, Guangxi, Guangdong	418634	129108.42	3.78	<0.01
2013	May.1 ~ Oct.31	Hainan, Guangxi, Guangdong	492978	110465.11	5.74	<0.01
2014	Apr.1 ~ Jul.31	Hainan, Guangxi, Guangdong	539360	111167.60	5.78	<0.01
2015	May.1 ~ Oct.31	Hainan, Guangxi, Guangdong	521140	121473.15	5.44	<0.01
2016	Apr.1 ~ Jun.30	Hainan, Guangxi, Guangdong	395469	79507.50	5.72	<0.01
2017	May.1 ~ Oct. 31	Hainan, Guangxi, Guangdong	598293	124496.50	6.53	<0.01
2018	May.1 ~ Sept.30	Shanghai, Jiangsu, Zhejiang	369979	120883.16	3.45	<0.01

### Geographical Detector

As shown in Table 4, meteorological factors were obviously associated with the incidence of HFMD. Precipitation had the strongest explanatory power for the spatial heterogeneity of the incidence of HFMD, with a q-value of 0.287 (*P*<0.001). Temperature, relative humidity, and hours of sunlight were also associated with the incidence rate of HFMD. The explanatory power q-value was 0.283, 0.212, and 0.015, respectively (*P*<0.001).

**Table 4:** Explanatory power of each meteorological factor on the incidence of HFMD.

	Precipitation	Temperature	Relative Humidity	Hours of Sunlight
*q*-value	0.287	0.283	0.212	0.015
*p*-value	<0.001	<0.001	<0.001	<0.001

The result of the interaction detector indicated that the interaction of any two meteorological factors has greater explanatory power than any single meteorological factor. Compared with their individual impact, they all showed an "enhance" effect. As shown in Table 5, taking relative humidity and temperature as examples, their *q*-values are 0.212 and 0.283, respectively. The *q*-value increased to 0.358 after considering the interactive effect of these two factors on HFMD incidence. The result suggested that relative humidity and temperature have a double-factor enhanced interactive association on the incidence of HFMD.

**Table 5:** The *q*-value of interactions between pairs of meteorological factors on the incidence of HFMD.

Meteorological Factors	Precipitation	Temperature	Relative Humidity	Hours of Sunlight
Precipitation	0.287			
Temperature	0.346	0.283		
Relative Humidity	0.315	0.358	0.212	
Hours of Sunlight	0.300	0.317	0.270	0.015

Results of the factor detector showed that socioeconomic and health resource factors were associated with the spatial heterogeneity of the incidence of HFMD (Table 6). Hospital beds per 10,000 persons had the strongest explanatory power, with a *q*-value of 0.078 (*P*<0.001). In addition, population density and GDP per capita were also associated with the incidence of HFMD, with explanatory power *q*-values of 0.074 and 0.070, respectively. The health technicians per 10,000 persons was not significantly associated with the incidence of HFMD (*P*>0.05).

**Table 6:** Explanatory power of each socioeconomic and health resource factor on the incidence of HFMD.

	Population Density	GDP Per Capita	Hospital Beds Per 10,000 Persons	Health Technicians Per 10,000 Persons
*q*-value	0.074	0.070	0.078	0.009
*p*-value	<0.001	0.040	<0.001	0.868

The interaction of any two socioeconomic and health resource factors has greater explanatory power than any single factor. Compared with their individual effect, they all showed a "nonlinear enhancement" effect. As shown in Table 7, the *q*-value of hospital beds per 10,000 persons was 0.078, which was increased to 0.230 after accounting for the interactive effect of population density on the HFMD incidence. As 0.230 is higher than the sum of 0.078(*q*-value of hospital beds per 10,000 persons) and 0.070(*q*-value of GDP per capita), the result indicated that hospital beds per 10,000 persons and GDP per capita have a nonlinear enhanced interactive association on the incidence of HFMD.

**Table 7:** The *q*-value of interactions between pairs of socioeconomic and health resource factors on the incidence of HFMD.

Socioeconomic and Health Resource Factors	Population Density	GDP Per Capita	Hospital Beds Per 10,000 Persons	Health Technicians Per 10,000 Persons
Population Density	0.074			
GDP Per Capita	0.222	0.070		
Hospital Beds Per 10,000 Persons	0.220	0.230	0.078	
Health Technicians Per 10,000 Persons	0.179	0.121	0.222	0.009

### Interrupted time series analysis

**Fig 6:**
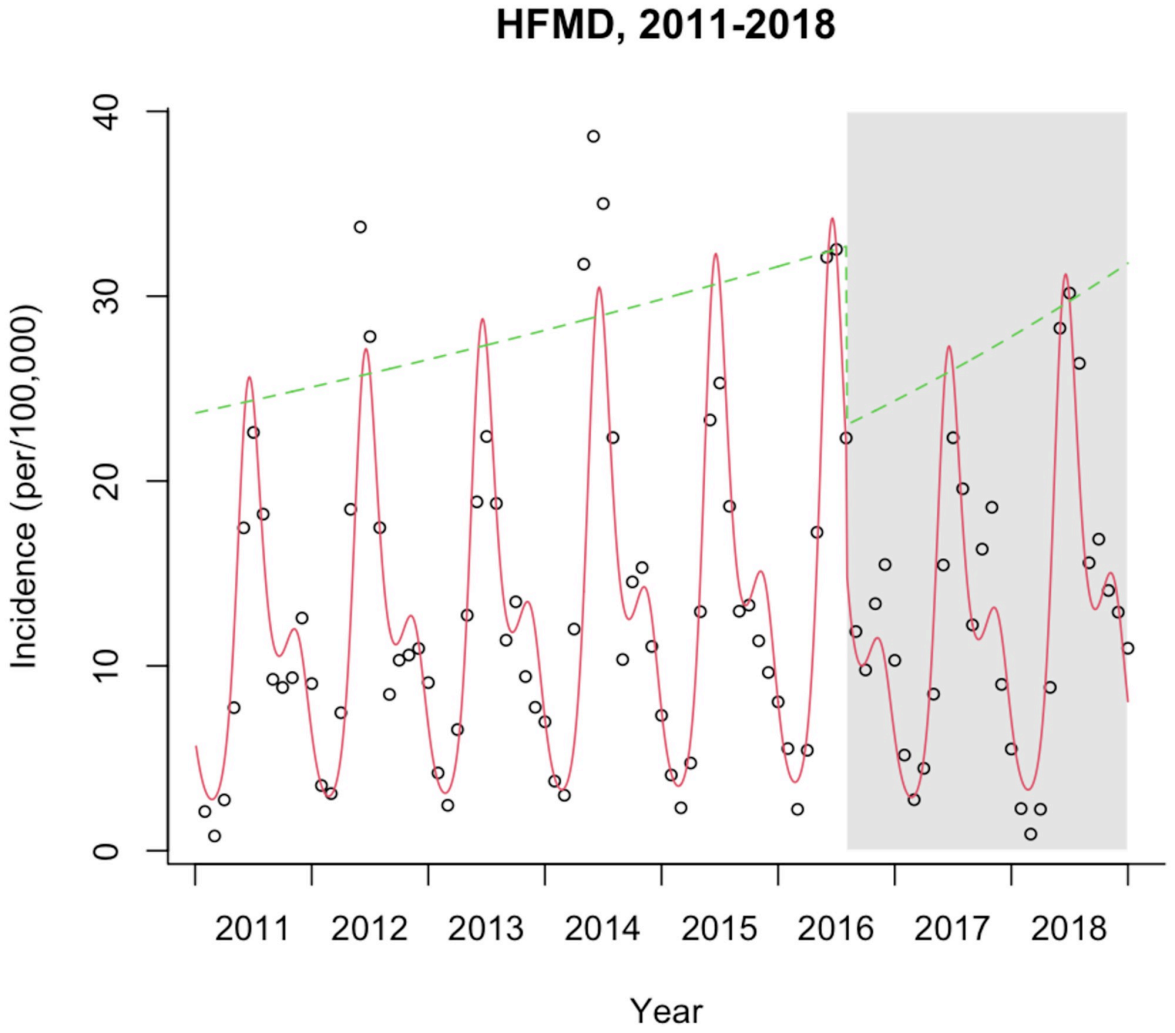
Scatter plot of monthly incidence and fitting curve of interrupted time series model in mainland China, 2011–2018. Dots represents monthly incidence; the red line is predicted trend based on the seasonally adjusted regression model; the green line represents the "de-seasonalized" trend.

This study conducted the interrupted time series analysis of HFMD cases in mainland China from 2011 to 2018. The scatter plot of monthly incidence and the fitted curve generated according to the model were drawn in Fig 6. The incidence of HFMD in mainland China showed an upward trend before the official introduction of the EV-A71 vaccine in most provinces. The slope of the model was 1.005 before the introduction of the vaccine(P<0.001) (Table 8). The instantaneous level of new cases decreased by 33.8% after introducing the vaccine (P<0.001). The change in the slope of the model after the introduction of the vaccine was 0.006 (P<0.001), which indicated that the incidence of HFMD after the introduction of the vaccine has increased faster than before.

**Table 8:** Interrupted time series analysis of HFMD cases in the most likely cluster, 2011–2018.

Province	Trends before the introduction of the vaccine		Immediate changes after the introduction of the vaccine		Changes of trends after the introduction of vaccines	
	*β*_1_	P-value	*β*_2_	P-value	*β*_3_	*P*-value
Mainland China	1.005	<0.001	-0.338	<0.001	0.006	<0.001
Guangdong	1.004	<0.001	-0.335	<0.001	-0.004	<0.001
Guangxi	1.005	<0.001	0.118	<0.001	-0.024	<0.001
Hainan	1.006	<0.001	-0.589	<0.001	-0.010	<0.001
Shanghai	1.000	<0.001	-0.659	<0.001	0.028	<0.001
Jiangsu	1.000	<0.001	-0.391	<0.001	0.033	<0.001
Zhejiang	1.002	<0.001	-0.757	<0.001	0.043	<0.001

We conducted the interrupted time series analysis of the HFMD cases in the 6 provinces (Guangdong, Guangxi, Hainan, Shanghai, Jiangsu, Zhejiang) of the most likely cluster from the Spatiotemporal clusters analysis (Table 8). The results indicated that, before introducing the HFMD vaccine, the incidence of HFMD showed an upward trend in Guangdong, Guangxi, Hainan, and Zhejiang. The incidence trends of HFMD were stable in Shanghai and Jiangsu. After the introduction of the vaccine, except for Guangxi, the instantaneous level of new HFMD cases decreased in another 5 provinces (*P*<0.001). After the introduction of the vaccine, the incidence of HFMD was no longer increasing in Guangdong (*P*<0.001) and showing a downward trend in Guangxi and Hainan (*P*<0.001). The incidence of HFMD showed an upward trend in Zhejiang, Shanghai, and Jiangsu. And the incidence of HFMD after the introduction of the vaccine has increased faster than before in Zhejiang (*P*<0.001). The scatterplot of monthly incidence and the fitted curve generated according to the model by province were plotted in S1 Fig.

## Discussion

To the best of our knowledge, there is limited evidence on the spatiotemporal distribution pattern of HFMD since the introduction of HFMD vaccines in mainland China and the assessment of the actual impact of the introduction of HFMD vaccines on HFMD incidence is lacking. In this study, we compared the spatiotemporal distribution pattern of HFMD incidence before and after the vaccine was launched in mainland China and analyzed the explanatory power of meteorological factors, socioeconomic factors, and health resource factors on the spatial heterogeneity of HFMD incidence. We also evaluated the impact of introducing HFMD vaccines on HFMD incidence. We have three important findings. First, the range of cluster time was between April and October. The most likely cluster of HFMD appeared in the southern coastal provinces (Guangxi, Guangdong, Hainan) from 2011 to 2017 and in the eastern coastal provinces (Shanghai, Jiangsu, Zhejiang) in 2018. Second, the spatial heterogeneity of HFMD incidence was mainly attributed to meteorological factors, which was also related to population density, GDP per capita, and hospital beds per 10,000 persons. Third, before the introduction of the EV-A71 vaccine, HFMD incidence was on the rise in the whole of mainland China. After introducing the EV-A71 vaccine, the instantaneous level of HFMD incidence decreased at the national level, and HFMD incidence trended downward in the southern coastal provinces and increased in the eastern coastal provinces.

This study found that the incidence of HFMD in mainland China showed an overall downward trend after 2014, and a substantial reduction in the incidence of HFMD was observed in the moments after the introduction of the vaccine, which may be related to HFMD vaccines launched against EV-A71 enterovirus in mainland China in 2016 [25, 45]. However, the interrupted time series analysis results indicated that, after the introduction of the vaccine, the incidence of HFMD showed an upward trend, and increased faster than before in mainland China. The incidence of HFMD in Mainland China was 169.41 per 100,000 in 2018, which was only lower than the incidence in 2014 and 2016, higher than Thailand’s incidence rate of 102.51/100,000 [46] and Malaysia’s incidence rate of 138.6/100,000 [47]. So, the incidence of HFMD was still at a high level in mainland China in 2018. There may be three reasons for this. On the one hand, the monovalent inactivated EV-A71 vaccines cannot provide protection against non-EV-A71 virus infections of HFMD [48]. On the other hand, due to the stable birth rate and no cross-protection of alternate serotypes, the previous HFMD epidemics did not reduce the susceptible persons in the population probably [49]. In addition, the vaccines of HFMD have not been included in the National Immunization Program in China, which means that parents need to vaccinate their children at their own expense and voluntarily. Hence, the vaccination coverage rate was not high [50]. A study found that the annual EV-A71 vaccination coverage rate of children under 5 years old ranged from 5.53% to 15.01% in Yunnan from 2016 to 2019 [51]. In Guangdong, the coverage rate of EV-A71 vaccination among the 6-month to 5-year children was 2.67% in 2016 and 10.07% in 2017 [52]. A study in Chengdu showed that the EV-A71 vaccination coverage rate should reach 94.0% to end the EV-A71-related HFMD transmission [53]. Therefore, efforts should be made to develop multivalent vaccines against major enteroviruses and increase the vaccination rate in the future.

The study results suggested the cyclical and seasonal pattern of the HFMD epidemics. The HFMD epidemic took two years as a cycle. This cyclical pattern was observed in many countries, and there was evidence that this cyclicality is related to the time required to accumulate enough susceptible children in the population [6, 54]. In this study, the seasonal pattern of HFMD was reflected in the peak incidence of twice a year, the first peak occurred in April to July, and the second peak occurred from September to November in mainland China. Seasonal patterns of HFMD have also been observed in other countries. HFMD usually breaks out from June to August in Thailand [55], cases of HFMD increased during the rainy season in Vietnam [56]. However, the seasonal patterns observed in temperate, tropical, and subtropical Asia are different [57]. In China, the seasonal characteristics of the south and the north were also inconsistent [58]. In mainland China, the first peak of each year may be related to climate factors such as temperature, humidity, and precipitation. Appropriate climatic conditions are conducive to the reproduction and survival of pathogens. Warm temperature promotes children’s activities and mutual contact, thereby promoting the transmission of pathogens. Although the temperature and humidity are still high in August, the school has summer vacation, which reduces the gathering and contact of students, so the incidence is reduced. September coincides with the end of summer and the beginning of autumn, so the temperature and humidity are still high. The second peak may be due to the opening of kindergarten in September to increase the contact between children, thereby increasing the possibility of cluster cases [19, 47, 59, 60].

We also found that children under 5 years are the main morbid population, accounting for 94.17% of all reported cases. The result was consistent with other research’s findings in other countries and China [1, 6, 46, 50]. The low incidence of children over 5 years old maybe because they have had HFMD before, thereby protecting them and their peers from HFMD [61]. In 2018, the incidence of children aged 5 and under was the lowest level in 10 years, which may be related to the increase in the cumulative coverage of vaccines [25]. Therefore, we call for attention to children in this age range, vaccinate them as soon as possible, and introduce the HFMD vaccine into the routine immunization program.

The global Moran’s I for 2011–2018 ranged from 0.419 to 0.547. The global spatial autocorrelation analysis results showed that the spatial distribution of HFMD cases was non-random in mainland China and tended to cluster at the provincial level, which was the same as the analysis result for 2008–2011 by Xiao [62]. Combined with the incidence distribution map (Fig 3), we found that the incidence of HFMD was quite different between the southern and northern of China. Overall, the incidence of south China was much higher than that in north China. This phenomenon has also been found in other studies [18, 19, 63]. The spatial heterogeneity of HFMD incidence may be mainly attributed to differences in meteorological factors among different regions, such as precipitation, temperature, relative humidity, and hours of sunlight. What’s more, the interaction of any two meteorological factors has greater explanatory power than any single meteorological factor. South China is mostly in high temperature, high humidity, and rainy weather conditions, which to a certain extent promotes the reproduction, survival, and spread of the pathogens of HFMD [18, 64]. Each year, there was only one peak incidence in late spring and early summer (May-July) in the northern region. At the same time, there were two peak incidences in April-June and September-November in the southern region [24, 58]. In terms of socioeconomic factors, we found that population density and GDP per capita impacted the incidence of HFMD. Previous studies have shown that socioeconomic factors play an essential role in the incidence of HFMD, with population density having the greatest impact on the spread of HFMD, and the incidence in economically developed areas is higher than in less developed areas [65–67]. The potential reason may be that the high population mobility in economically developed regions, in the interaction with high population density, promoted the spread of HFMD. Moreover, compared with population density and GDP per capita, hospital beds per 10,000 persons ha a greater explanatory power for HFMD incidence. The result is consistent with the findings in Shandong Province [23]. It suggests that the healthcare level may play an essential role in controlling the spread of HFMD.

The most likely cluster appeared in southern coastal provinces (Guangxi, Guangdong, Hainan) from 2011 to 2017. The results of local spatial autocorrelation analysis and spatiotemporal clusters analysis indicated that the southern coastal provinces (Guangxi, Guangdong, Hainan) were the areas with a high incidence of HFMD in China. Their incidence had been at the highest level in mainland China for many years, the same as previous research results [18, 68, 69]. The climate of these provinces is characterized by short and mild winter, while summer is long, hot, and very humid. In addition to climatic factors, the high incidence in southern coastal provinces may also be due to the high population density, strong population mobility, developed economy, and more frequent communication and close contact between individuals, which led to a higher risk of spreading HFMD [47, 60, 68–70]. The cluster time ranged between April and October, which was consistent with the peak time of HFMD in mainland China [1]. In 2017, the relative risk of the most likely cluster (Hainan, Guangxi, Guangdong) was 6.53, which was higher than before. Because the incidence of HFMD had decreased in other provinces, while the incidence of Hainan, Guangxi, and Guangdong were still at a high level, the low coverage rate of the vaccine may be one of the reasons. In 2017, the cumulative coverage rate of EV-A71 vaccine among 6-month to 5-year children was 9.26% in Guangxi [25] and 10.07% in Guangdong [52], while it was 18.94% in Yunnan [51] and 15% in Beijing [71]. In addition, some studies have found that non-EV-A71 enteroviruses were the dominant serotype in Guangxi in 2017, while the monovalent EV-A71 vaccine cannot generate cross-immunity against other enterovirus infections [25, 60, 72]. The interrupted time series analysis indicated that, after the introduction of the EV-A71 vaccine, the incidence of HFMD was lower than before in Guangdong, Guangxi, and Hainan. The incidence of HFMD was no longer increasing in Guangdong and showing a downward trend in Guangxi and Hainan In 2018, the southern coastal provinces (Guangxi, Guangdong, Hainan) were no longer the most likely cluster. The decline in the incidence of HFMD was large in these three provinces, and the incidence had reduced to the lowest level during the study period, which was 258.10 per 100,000 in Hainan and 354.28 per 100,000 in Guangxi, 265.23 per 100,000 in Guangdong. This result may be related to the increase in vaccine coverage. Since Guangxi implemented comprehensive prevention and control measures focusing on promoting the EV-A71 vaccine among susceptible populations, the vaccine coverage rate of susceptible children had approached 30% in Guangxi in 2018, and the EV-A71-related incidence had declined [25, 72]. The same finding was also found in the study of Guangdong [60, 73]. For this reason, it is necessary to expand the HFMD vaccination program.

According to the results of spatiotemporal clusters analysis, we found that three eastern coastal provinces (Shanghai, Jiangsu, Zhejiang) were included in secondary cluster 1 from 2011 to 2016. The relative risk of this cluster was only second to the most likely cluster. This result indicated that these provinces were areas with a high incidence of HFMD, and they had a higher incidence than the northern and inland provinces. These provinces have a subtropical climate, so they are tended to be hotter and more humid. Furthermore, they have a higher population density and a larger migratory population. These factors are the main reason for the high incidence of HFMD [74]. In 2018, these provinces (Shanghai, Jiangsu, Zhejiang) constituted the new most likely cluster, and the cluster time ranged between May and September. We also discovered that the new High-High cluster area appeared in Jiangsu in 2018 through local spatial autocorrelation analysis. However, previous research by Chao Wang found that the most likely cluster was observed in the Shandong, Hebei, Henan, and Shanxi provinces in 2009, and were located in Guangdong, Guangxi, and Hainan provinces in 2010–2012 [24]. Combined with our findings, it can be demonstrated that the spatiotemporal clustering pattern of HFMD had changed in 2018. The results suggested that the incidence of HFMD in Jiangsu and its neighboring Shanghai and Zhejiang was higher than that of other provinces in 2018, which was found in other studies [75, 76]. However, the incidence of the previous most likely cluster (Hainan, Guangxi, Guangdong) had decreased, so the eastern coastal provinces (Shanghai, Jiangsu, Zhejiang) became the new most likely cluster. According to the interruption time series analysis, after the introduction of the EV-A71 vaccine, the incidence of HFMD showed an upward trend in Zhejiang, Shanghai, and Jiangsu. In 2018, the incidence in Zhejiang and Jiangsu reached the highest value after the vaccine was launched, which was 436.02 per 100,000 in Zhejiang and 237.50 per 100,000 in Jiangsu, and Zhejiang had the highest incidence in the country. EV-A71 vaccine coverage rate had increased year by year to 24.05% in Zhejiang, higher than 19.4% in Guangdong [77]. The proportion of EV-A71 positive cases declined from 22.6% in 2017 to 3.3% in 2018 in Zhejiang [78]. Another study found that CV-A16 and CV-A6 became the primary pathogens of HFMD in Jiangsu, and the cases caused by these two pathogens accounted for more than 96% of all cases [79]. Therefore, the rise in the incidence of HFMD may be related to other serotypes becoming dominant after the introduction of the EV-A71 vaccine. Even large-scale vaccination of the EV-A71 vaccine would not greatly reduce the number of cases [25, 79]. This result suggested that the dominant pathogens in different regions and at different times are various. Therefore, it is necessary to monitor the enterovirus genotypes of HFMD cases in various places and promote HFMD multivalent vaccines.

Compared with studies in various provinces [68, 69, 80], we found that clusters were not only distributed in one province but also cross-linked to multiple provinces, which showed the importance of cooperation between provinces to prevent and control the spread of HFMD [24]. This strong spatial and temporal correlation of HFMD was also found in other studies, which may be related to meteorological factors. Because meteorological factors promote the prevalence of HFMD, the meteorological conditions in adjacent areas and adjacent time points are similar [74].

The southern inland province Hunan was included in the High-High cluster areas in 2014 and 2016–2018. Although Hunan had not appeared in the most likely cluster, the results of spatiotemporal clusters analysis showed that Hunan was one of the high-risk areas for HFMD. It was observed to have a high incidence, which was also found in other studies [81]. Because Hunan has a subtropical monsoon climate with abundant heat, concentrated rainfall, and high humidity, in addition to high population density and high levels of migration, both suitable natural and social environments have promoted the development and spread of enteroviruses in Hunan [80, 82]. This result suggested that the HFMD epidemic in Hunan was worthy of attention.

For the prevention and control strategy of HFMD, we make the following suggestions: First, the prevention and control of HFMD should focus on eastern and southern coastal provinces in mainland China, especially from April to September each year. Attention should also be paid to the southern inland provinces. Moreover, regarding the strong spatial and temporal association of HFMD in neighboring provinces, the provinces should strengthen cooperation and joint control to avoid a widespread HFMD across provinces. In addition, it is necessary to advance the EV-A71 vaccination plan and expand the vaccine coverage, especially in coastal areas of China. As the dominant pathogens have changed, future research should analyze the spatial-temporal distribution characteristics of different pathogens of HFMD and develop multivalent HFMD vaccines as soon as possible.

The main advantage of our research is using Chinese CDC’s disease surveillance data, so the authenticity and completeness of the data are guaranteed. In addition, our study used spatiotemporal clusters analysis that combines time and space, revealing the temporal and spatial distribution patterns of HFMD at the province level in mainland China. We set 15% of the risk population as the maximum cluster size in the analysis, avoiding the most likely cluster containing low-risk regions and thus larger than the actual cluster. In this study, the data of HFMD cases were scanned year by year, and the trend of the most likely cluster could be found. As far as we know, it is the first study to analyze the spatiotemporal clusters of HFMD in the whole of mainland China after the HFMD vaccines were launched in China. Furthermore, we used interrupted time series analysis to assess the actual impact of the introduction of the EV-A71 vaccine on HFMD incidence.

The limitations of this study are worth mentioning. First, although Chinese CDC disease surveillance data could ensure the authenticity and completeness of the data to a certain extent, it may not avoid the differences in the quality of case reports in the surveillance systems of different regions and the underreporting of cases caused by mild cases that do not go to the hospital for treatment. In addition, the outbreak of COVID-19 in 2019 may change the spatial and temporal distribution pattern of HFMD in China. However, we have not obtained HFMD data after 2019. The incidence data of HFMD was at the provincial level. More accurate results may be attained if using smaller spatial scale (such as district, county) information. This study set 15% of the total population as the maximum cluster size based on previous research experience. Using MCS-P to select the optimal maximum cluster size can improve the performance of scan statistics [38]. But it does not apply to the data in this study. On the other hand, the spatial scanning window used to detect the cluster was circular, while the geographic shape was irregular, which may not represent the actual shapes of the clusters. What’s more, because we did not have access to pathogen data of HFMD cases, the spatiotemporal clusters analysis of HFMD pathogens was not possible. Substantial evidence indicated that other virus serotypes became dominant after the introduction of the EV-A71 vaccine. Future research should focus on the pathogen agents of HFMD.

## Conclusions

From 2011 to 2018, the high-risk areas of HFMD were mainly located in areas with suitable climatic conditions for the survival and spread of the virus, high population density, and strong population mobility. After introducing the EV-A71 vaccine, the level of HFMD incidence decreased at the national level, but the impact of the introduction of the EV-A71 vaccine on the incidence of HFMD was heterogeneous in different areas. The prevention and control policies of HFMD should be adapted to local conditions in different provinces. It is necessary to advance the EV-A71 vaccination plan, expand the vaccine coverage and develop multivalent HFMD vaccines as soon as possible.

## Supporting information

S1 Fig Scatter plot of monthly incidence and fitting curve of interrupted time series model in the most likely cluster, 2011–2018. Dots represents monthly incidence; the red line is predicted trend based on the seasonally adjusted regression model; the green line represents the "de-seasonalized" trend.
(TIF)
