# Supplementary figures and images for: Spatiotemporal cluster patterns of hand, foot, and mouth disease at the province level in mainland China, 2011–2018
Source: PLoS One. 2022 Aug 22;17(8):e0270061. doi: 10.1371/journal.pone.0270061 (PMC9394824; doi:10.1371/journal.pone.0270061)

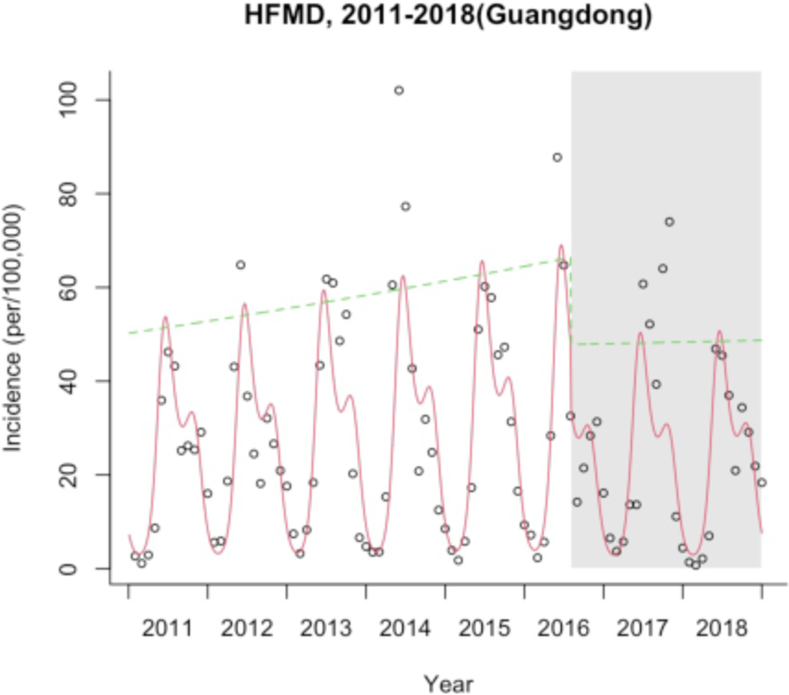

Supplement: S1 Fig — Dots represents monthly incidence; the red line is predicted trend based on the seasonally adjusted regression model; the green line represents the "de-seasonalized" trend. (TIF) [file pone.0270061.s001.tif]
